# Peri-Arterial Sympathectomy
*Communicated to the Bath and Bristol Branch of the British Medical Association, October 31st, 1928.


**Published:** 1928

**Authors:** E. W. Hey Groves

**Affiliations:** Professor of Surgery in the University of Bristol; Senior Surgeon, Bristol General Hospital


### PERI-ARTERIAL SYMPATHECTOMY.*

BY

E. W. Hey Groves, M.S., F.R.C.S.,

*Professor of Surgery in the University of Bristol;*
*Senior Surgeon, Bristol General Hospital.*

---

When I was invited to submit a paper to this meeting, I had no hesitation in choosing this subject, because I had just returned from an extraordinarily interesting and stimulating visit to the clinic of Leriche at Strasbourg. Professor Leriche, who was the successor of Ollier at Lyons, is doing pioneer work on the subject of sympathectomy, a subject which concerns a large number of general diseases which come primarily under the care of the man in general practice, and which, for ultimate analysis and treatment, ought to be seen by the physician and surgeon in consultation.

Peri-arterial sympathectomy as an operation is comparatively simple. It runs the risk, however, like so many other procedures, of being applied indiscriminately and perhaps carelessly, and there are therefore those who are inclined to discount the value of the operation. One sometimes meets people who say they have employed it in two or three cases, but do not seem to attach much importance to it. We were greatly impressed by the amount of work done at Strasbourg on the subject, both experimental and clinical, and by its careful nature, particularly from the medical and neurological points of view. A great deal

* Communicated to the Bath and Bristol Branch of the British Medical Association, October 31st, 1928.

of it was beyond the understanding of the mere surgeon. It was pointed out that a detailed diagnosis must always precede the application of treatment.

The actual operation itself consists of a careful stripping of the adventitia of the main artery for a distance of 8 to 10 cm. Obviously, the operation may be extremely simple, but on the other hand it may be difficult, by reason of adhesion of the sheath to the vessel. In a case I myself recently operated upon, a man with a chronic ulcer of the leg which refused to heal, the origin of which was found to be traumatic, the artery was quite clearly the subject of surrounding inflammation, which confirmed the idea that in this case there had been a definite irritation in the perivascular tissues. The operation consists of the exposure of either the brachial or the femoral artery, and stripping off its adventitia for a distance of about 8 to 10 cm. The whole circumference of the artery has to be stripped in order to remove the adventitia which has adhered to the fibrous tissues and muscles. It is a matter of great importance to avoid any injury to the media. If the media is injured, dilatation and rupture of the artery may follow, but it ought not to be difficult to avoid this danger. Leriche uses a curious little instrument, something between a scalpel and a probe.

The first and most important point to make clear is that the result of the operation is not merely a local but also a general one. Always there appears a rise of temperature in the affected limb, a general vaso-dilatation and some degree of leucocytosis. Leriche holds that it is in the nature of a reflex manifestation. His evidence for this belief is that the vaso-dilatation affects other parts of the body, especially the opposite limb as well as that operated upon. The rise of temperature and leucocytosis do not usually last more than two or three weeks, but the other results are much more durable.

For simplicity's sake, the indications for the operation may be stated under five headings, although of course this division might be made much more elaborate: (1) pain, (2) vasomotor phenomena, (3) injuries or diseases of the arteries and veins, (4) chronic and trophic ulcers, (5) bone and joint disorders.

I will speak of these all in order, mentioning, where occasion allows me, the operations which we actually saw Leriche perform.

A point which strikes one as interesting was his application of sympathectomy to the alleviation of pain. Causalgia is a painful neurosis of the limb, often seen as a sequela of war wounds of the limbs, and characterised by pain like a burning, gnawing heat. It was as an experiment in 1917 that Leriche treated a case of causalgia (his first application of this procedure), and the successful result sitmulated him to go on developing the method. Other forms of neurosis following injury are open to this treatment, provided, of course, that there is no definitely local distinct cause for pain. He has, however, found that what he describes as an "ascending neuritis" of traumatic origin is out of the scope of this treatment. We all know the type of case in which the patient has had one amputation after another in the vain attempt to cure. Leriche gives an account of the case of a man who had had his thumb amputated at Lyons, his wrist amputated at Marseilles, his elbow at Dijon, and his shoulder in Paris.

The painful stump is another condition which is amenable to the application of peri-arterial sympathectomy. There is the type not presenting an adherent scar or neuroma, but with paroxysms of pain, somewhat of the type of causalgia. This is the type of painful stump in which sympathectomy is most valuable. If the main artery be found to be occluded, instead of peri-arterial sympathectomy, the occluded part of the artery should be excised. There is a second type which is called the "phantom limb," in which the pain is of central origin and quite unamenable to sympathectomy.

The last group was the most interesting to us, because it was most novel—the application of the method to certain cases of intractable abdominal pain. One can readily understand how an enthusiast might exploit this, if the idea is taken up. We actually saw Leriche do a sympathectomy for a case of very painful carcinoma of the rectum. Unfortunately, we are all familiar with cases for which the only remedy is morphine ; or a division of the sensory tracts of the spinal cord, or possibly a rhizotomy, both of which are dangerous operations. Leriche opened the abdomen, the patient being in the Trendelenberg position, exposed the aorta, divided the nerve fibres round the bifurcation which runs down to the pelvis. It appears to be a simple and easy operation which is practically devoid of any serious risk, and can therefore be very well applied to this type of disease, even if a more desperate operation has to be resorted to later. Another indication for the operation was the severe spasm of dysmenorrhœa, in which other remedies had failed.

The next group to which sympathectomy may be applied consists of the vasomotor syndromes, such as Raynaud's disease, with such diseases of the skin as sclerodermia. We were struck by the discrimination which Leriche exercises in distinguishing between the types of the diseases. There was the typical form of Raynaud's disease in which the fingers go white. Those are the cases in which peri - arterial sympathectomy gives the greatest relief, and apparently prevents gangrene. For those types of Raynaud's disease in which the fingers are blue and there is obviously a chronic condition of vaso-dilatation sympathectomy gives no good result.

Thirdly, there is the group of definite injuries or diseases of the arteries and veins. We saw several cases of thrombo-angeitis obliterans, one of whom was operated on—a young man with thrombosis at the bifurcation of the brachial artery. In those cases of what Leriche calls "juvenile traumatic arteritis" he has shown that it is not sufficient merely to strip off the adventitia, but that it is more satisfactory to do an excision of the affected part of the artery. He holds that sympathectomy does not give such good results as excision. He showed us several cases.

Fourthly, there are the chronic and trophic ulcers. Probably this is the indication which if it is properly understood will afford to the operation its most valuable opening, as we all know what a hopeless thing a chronic congestive ulcer is, and what a help it would be if we could find any short cut to its cure. Leriche showed a number of cases in which skin grafting and other methods had failed, but where much more rapid healing took place after sympathectomy.

The last group of cases was to me personally the most interesting of all, because it opened up a still wider field which one had never thought of. I refer to bone and joint disorders. There are certain types of injury to the limbs which resist simple treatment, leaving a thickening round the joint, and resembling a fibrous ankylosis. The special point of diagnosis is that the X-rays show a notable decalcification of the bones, and in such types of cases treatment by sympathectomy produces an improvement of the circulation and relief of the pain in the limb in a remarkable degree. Another joint disorder for which it has been used is that of chronic tuberculosis. Lastly, in this group, bony union is greatly hastened by sympathectomy. Clinically it has proved useful, in the treatment of ununited fractures. Union becomes more quickly consolidated owing to the greater cellular proliferation which takes place in the area. In ischæmic paralysis it seems quite reasonable that the method should be valuable. Here again, if he finds the artery has been damaged, then Leriche chooses excision.

I have given you rather a hurried summary of what was to me an extremely interesting and suggestive exposition, and I only hope that these few ideas will be as stimulating to you as they were to me—it may be to adverse criticism, but it must be to thought.

